# Fetoscopic repair of myeloschisis: optimizing the three-layer closure

**DOI:** 10.3171/2026.1.FOCVID25217

**Published:** 2026-04-01

**Authors:** Matthieu D. Weber, Ryan B. Juncker, Eric A. Sribnick, Karen A. Diefenbach, Adolfo Etchegaray, Jeffrey R. Leonard, Oluseyi K. Ogunleye, Jonathan A. Pindrik, Oluyinka O. Olutoye, Albert M. Isaacs

**Affiliations:** 1 College of Medicine, The Ohio State University, Columbus;; 2 Department of Neurological Surgery, The Ohio State University, Columbus;; 3 Department of Neurological Surgery, Nationwide Children’s Hospital, Columbus;; 4 Department of Pediatric Surgery, Nationwide Children’s Hospital, Columbus;; 5 Division of Fetal Medicine, Nationwide Children’s Hospital, Columbus; and; 6 The Fetal Center, Nationwide Children’s Hospital, Columbus, Ohio

**Keywords:** myeloschisis, spina bifida, fetoscopy, multilayer, repair, pediatric, neurosurgery, fetal surgery

## Abstract

A 25-year-old female presented at 24 weeks and 2 days of gestation with suspected myeloschisis on fetal anatomical survey. Imaging demonstrated an L4–S4 defect, ventriculomegaly, and Chiari type II malformation. Closure remains one of the least standardized components of fetoscopic repair, although small studies have suggested that three-layer repair may reduce CSF leakage, shorten maternal hospitalization, decrease revision rates, and improve Chiari malformation outcomes. The patient underwent a three-port fetoscopic repair with three-layer closure. The authors showcase their approach and advocate for closure technique standardization and its correlation with long-term neurological outcomes in the post–Management of Myelomeningocele Study (MOMS) trial era.

The video can be found here: https://stream.cadmore.media/r10.3171/2026.1.FOCVID25217

## Transcript

This video represents the three-port fetoscopic three-layer repair of a fetus with myeloschisis.

### 0:26 History of Present Illness.

This is the case of a 25-year-old G7P4024 female presenting at 24 weeks and 2 days of gestation with concern for spina bifida seen on anatomical survey. She reported regular fetal movement, and her current pregnancy had so far been uncomplicated. She has a medical history of chronic hypertension and an MRI-incompatible metallic foreign object from a previous gunshot wound. She has an obstetric history of four spontaneous vaginal deliveries, prior gestational diabetes, and a child with spina bifida occulta. She had a fluorescence in situ hybridization test performed after amniocentesis, which was normal. Her fetal ultrasound imaging showed a 2.5-cm flat lumbosacral defect, hindbrain herniation, and ventriculomegaly. She was counseled on and consented to a fetoscopic repair of the fetal lumbosacral defect with the goals of preserving neural elements, as well as improving postnatal lower extremity function, hydrocephalus, and hindbrain herniation.

### 1:22 Background and Rationale.

Fetoscopic spina bifida repair is a relatively new technique that seeks to achieve the neurological benefits shown in the MOMS trial while reducing maternal morbidity associated with open repair.^1–4^ Fetoscopic repair has been shown to prevent uterine morbidity, allow for vaginal delivery, and increase gestational age at delivery when compared to hysterotomy-based techniques.^5^ Although port placement and uterine access have improved, repair and closure of the placode and soft tissues remain technically challenging, with variability in materials, layer sequence, and techniques used for closure.^6–8^ An incomplete or nonwatertight closure can lead to CSF leaks, skin dehiscence, and increased revision rates.^8,9^ We present our approach to fetoscopic repair with an emphasis on evidence-based techniques for defect closure.

### 2:05 Preoperative Imaging.

The fetal ultrasound revealed a 25-week 1-day-old fetus in the 61st percentile for growth. Anatomical survey revealed an L4–S4 spinal defect without overlying membranes concerning for myeloschisis. Imaging of the cranium revealed ventriculomegaly with left and right ventricles measuring 12.3 and 9.5 mm, respectively. The posterior fossa showed hindbrain herniation concerning for Chiari type II malformation. Motor function of bilateral S1 levels appeared intact. Additionally, a single umbilical artery with normal flow on Doppler evaluation and a normal echocardiogram were observed.

### 2:43 Summarized Steps of the Procedure.

The three-port fetoscopic repair of myeloschisis was performed at 26 weeks and 1 day of gestation.^10^ Our group aims to operate within the MOMS trial window whenever possible; however, late referral and completion of standard preoperative evaluation in this case required multidisciplinary consensus to operate 1 day outside of that window. For the operation, the patient was placed in the lithotomy position. Uterine relaxation was achieved with a 4-g magnesium bolus at the start of the case and 2-g/hr infusion throughout the case. A Pfannenstiel incision was used to dissect down to and expose the uterus. The uterus was exteriorized, and ports were placed under ultrasound guidance. The repair was performed in a three-layer fashion, ensuring watertight closure. The dural and myofascial layers were closed first, followed by fetal skin closure. After the repair, the ports were closed, the uterus repositioned within the abdomen, and the maternal soft tissues were closed.

### 3:37 Maternal Skin Incision and Soft Tissue Dissection.

After initial incision, a combination of electrocautery and blunt dissection through the subcutaneous tissues was used to access the superficial rectus sheath, which was cut transversely to expose the rectus muscle. The dissection was carried superiorly, and the rectus was then split in the midline. The parietal peritoneum was then incised, exposing the uterus.

### 3:57 Uterus Exteriorization and Port Placement.

The uterus was exteriorized, and under ultrasound guidance the fetus was positioned with the back facing upward. Membranes were anchored using 2-0 absorbable stitches in box formation, and two 12-Fr and one 10-Fr fetoscopic ports were inserted using the ultrasound-guided Seldinger technique. A third port, not shown here, was triangulated and placed under endoscopic visualization. Fifty ml of amniotic fluid were withdrawn to improve visualization, and the uterus was insufflated with warm carbon dioxide to a maximum pressure of 7 mm Hg. Once fetal anesthesia and stable fetal echocardiography were confirmed, we proceeded with placode mobilization.

### 4:34 Placode Exposure and Zona Epitheliosa Dissection.

Endoscopic view showed a flat lumbosacral defect with a broad zona epitheliosa surrounding the placode. A small opening was made in the zona epitheliosa, which was extended using scissors. This plane was followed circumferentially around the defect to separate the epithelial rim from the placode and release it. A combination of sharp and blunt spreading dissection was used to achieve a complete dissection of the placode from the epithelial rim. Special attention was paid to cauterize vessels in the periphery to maintain hemostasis. We made sure to dissect all nonneural and epithelial tissue from the placode surface to prevent future dermal inclusion. We show the result prior to repair—the placode has been circumferentially mobilized, and the placode and associated nerve roots drop freely into the spinal canal.

### 5:23 Skin Undermining and Myofascial Flap Creation.

To prepare for the repair, we undermined the skin overlying the myofascial tissue by dissecting the plane between the two layers. Using the electrocauterized hook, we then raised bilateral myofascial flaps. These flaps spanned the entire length of the defect to ensure watertight closure.

### 5:49 Dural, Myofascial Repair.

For the dural repair, a 2.8 × 0.8–cm piece of dural substitute graft was positioned overlying the defect. Because myeloschisis often lacks a safe dural edge, we avoided anchoring sutures and, under continuous visualization, stabilized an intracanally placed graft with the myofascial closure. We then used an interrupted absorbable stitch within the myofascial tissue to secure the underlying graft in position and hold the myofascial layers for closure. A 5-0 barbed suture was then used to close the raised myofascial flaps. A running technique is used for this step, progressively tightening the suture to the desired tension. We show the completed myofascial repair prior to skin closure.

### 6:43 Skin Repair.

The fetal skin was then closed primarily using a running locking technique with a 5-0 absorbable suture. Given the span and location of the defect, which had the potential to complicate a tension-free closure, skin reapproximation was started at both the caudal and cranial ends. The two pieces of suture were tied in the middle, progressively tightening until good edge-to-edge contact was observed. We were able to achieve a strong closure with good approximation without evidence of substantial tension at the suture line or the need for relaxing incisions.

### 7:20 Port Closure and Soft Tissue Closure.

Following skin closure, the carbon dioxide was evacuated, and warm lactated Ringer’s solution was infused to restore amniotic volume. Intra-amniotic clindamycin was also administered at this time. Ports were closed in two layers with both superficial and deep-buried sutures. A site of chorioamniotic membrane separation created during port insertion was repaired in a similar way. Maternal soft tissues were closed in a standard multilayered fashion.

### 7:47 Postoperative Follow-Up.

The patient did well postoperatively. She had intermittent contractions on postoperative day 3, which were well managed with terbutaline as needed, in addition to scheduled nifedipine and indomethacin. Her stay was otherwise uneventful, and she was discharged on postoperative day 5 with scheduled multidisciplinary follow-ups with her neurosurgery and fetal-maternal medicine teams. Her follow-up on postoperative day 12 showed normal interval growth of the fetus, stable ventriculomegaly, and intact S1 motor function bilaterally.

### 8:15 Postnatal Follow-Up.

Postnatal MRI obtained on day of life 3 demonstrated no significant ventricular enlargement, reversal of hindbrain herniation, no pseudomeningocele, and a well-healed repair site with granulation tissue and no wound dehiscence.

### 8:28 Conclusions.

In conclusion, the defect closure remains one of the least standardized steps of fetoscopic spina bifida repair, with institutional variations in graft selection, layer sequence, and suturing strategy.^6–8^ A three-layer reconstruction with complete placode mobilization offers improved mechanical protection of the placode and, when feasible, may reduce rates of postnatal CSF leakage, shorten maternal length of stay, decrease postnatal revision rates, and higher rates of hindbrain herniation reversal when compared to single-layer closure.^9^ Watertight dural closure using a synthetic matrix patch can separate the placode from amniotic fluid and may minimize adhesion formation. In contrast to myelomeningocele, where the CSF-filled sac and residual membranes provide some separation of the placode, flat defects often complicate removal of epithelial tissue from the placode and nerve roots due to adherence. In myeloschisis, this step often requires more extensive circumferential dissection to reduce the risk of dermal inclusion and tethering. In these lesions, since the placode is typically flush with surrounding soft tissue, significant myofascial mobilization is also required to obtain a watertight reconstruction. Three-layer closure may also reduce rates of tethered cord formation when compared to single-layer closure, though longer follow-up times are needed to determine true rates.^9^ Though it is technically demanding, especially for larger defects like the one seen in our case, myofascial reapproximation can reinforce the dural repair and decrease tension when closing skin. In this case, absence of preexisting skin laxity around the defect required significant undermining and tension-distributing suture techniques, such as running locking closure with two or more suture anchor points, which were used to facilitate primary skin closure without relaxing incisions. To establish best practices, there must be continued efforts across pediatric centers to standardize and correlate closure technique with long-term neurological outcomes.

## Disclosures

K. Diefenbach reported consulting fees from Hologic and Asensus outside the submitted work. O. Olutoye reported grants from Medtronic and Mallinkrodt outside the submitted work.

## Author Contributions

Primary surgeon: Etchegaray, Isaacs. Assistant surgeon: Diefenbach, Leonard, Ogunleye, Olutoye. Editing and drafting the video and abstract: Weber, Juncker, Sribnick, Diefenbach, Etchegaray, Leonard, Ogunleye, Pindrik, Isaacs. Critically revising the work: Weber, Juncker, Sribnick, Etchegaray, Ogunleye, Pindrik, Isaacs. Reviewed submitted version of the work: Weber, Juncker, Sribnick, Diefenbach, Etchegaray, Leonard, Pindrik, Olutoye, Isaacs. Approved the final version of the work on behalf of all authors: Weber. Supervision: Sribnick, Etchegaray, Leonard, Isaacs.

## Supplemental Information

### Patient Informed Consent

The necessary patient informed consent was obtained in this study.
